# The assessment of possible gender-related effect of endogenous striatal alpha-tocopherol level on MPTP neurotoxicity in mice

**DOI:** 10.1016/j.heliyon.2020.e04425

**Published:** 2020-07-11

**Authors:** Nikolett Nánási, Gábor Veres, Edina K. Cseh, Diána Martos, Levente Hadady, Péter Klivényi, László Vécsei, Dénes Zádori

**Affiliations:** a Department of Neurology, Interdisciplinary Excellence Center, Faculty of Medicine, Albert Szent-Györgyi Clinical Center, University of Szeged, Szeged, Hungary; b MTA-SZTE Neuroscience Research Group, Szeged, Hungary

**Keywords:** Parkinson's disease, MPTP, Tocopherol, Dopamine, Striatum, Gender, Chemistry, Analytical chemistry, Biological sciences, Neuroscience, Neurotoxicology, Biochemistry, Antioxidant, Neurology

## Abstract

Several studies supported an increased vulnerability of males regarding Parkinson's disease (PD) and its animal models, the background of which has not been exactly revealed, yet. In addition to hormonal differences, another possible factor behind that may be a female-predominant increase in endogenous striatal alpha-tocopherol (αT) level with aging, even significant at 16 weeks of age, previously demonstrated by the authors. Accordingly, the aim of the current study was the assessment whether this difference in striatal αT concentration may contribute to the above-mentioned distinct vulnerability of genders to nigrostriatal injury.

Female and male C57Bl/6 mice at the age of 16 weeks were injected with 12 mg/kg body weight 1-methyl-4-phenyl-1,2,3,6-tetrahydropyridine (MPTP) 5 times at 2 h intervals or with saline. The levels of some biogenic amines (striatum) and αT (striatum and plasma) were determined by validated high performance liquid chromatography methods.

Although the results proved previous findings, i.e., striatal dopamine decrease was less pronounced in females following MPTP treatment, and striatal αT level was significantly higher in female mice, the correlation between these 2 variables was not significant. Surprisingly, MPTP treatment did not affect striatal αT concentrations, but significantly decreased plasma αT levels without differences between genders.

The current study, examining the possible role of elevated αT in female C57Bl/6 mice behind their decreased sensitivity to MPTP intoxication for the first time, was unable to demonstrate any remarkable connection between these 2 variables. These findings may further confirm that αT does not play a major role against neurotoxicity induced by MPTP.

## 1 Introduction

Although neurodegenerative disorders, including Parkinson's disease (PD), may differ in clinical and pathological characteristics, their pathomechanisms may involve some common features, such as glutamate excitotoxicity, mitochondrial dysfunction and reduced antioxidant capacity [1]. Regarding the antioxidant aspects, strong evidence suggest that α- and γ-tocopherols have important role in antioxidant protection in the central nervous system (CNS) due to its lipid-rich structure [2, 3, 4, 5]. Numerous studies assessed age- and gender-related differences in α-tocopherol (αT) levels in serum/plasma samples of healthy individuals [6, 7] and furthermore, the influencing effect of dietary tocopherol intake on PD-related parameters, including those studies that focused on the achievement of neuroprotection via the administration of exogenous αT [1, 3, 8, 9, 10, 11, 12, 13, 14, 15, 16]. However, the results of studies using exogenous tocopherol supplementation are controversial; some demonstrated that vitamin E intake may be beneficial regarding disease evolution [12, 14], whereas others found no effect on it [3, 9, 10, 13]. Long-term αT and ascorbate treatment effectively delayed the need for the use of levodopa (L-DOPA) by an average of 2.5 years [17] when they were applied in combination at considerably high doses (3200 IU αT and 3000 mg ascorbic acid per day) compared to daily 2000 IU αT in the DATATOP trial which could not demonstrate any beneficial effect of daily vitamin E supplementation on delaying the onset of disability in PD [9]. The possible explanations for these findings may be that the ability of dietary αT to enrich cellular membranes, especially in the CNS, is limited and needs the administration of considerably high doses for a long time [18]. Furthermore, the form of the administered αT may count as well [18]. Accordingly, the achievement of the enrichment of mitochondria with protective levels of αT in the striatum and substantia nigra is quite challenging.

Regarding experimental models of PD, probably the administration of 1-methyl-4-phenyl-1,2,3,6-tetrahydropyridine (MPTP) toxin with mitochondrial respiratory chain complex I inhibitory properties is the most widely applied [19, 20]. The active metabolite of this toxin, 1-methyl-4-phenylpyridinium ion (MPP^+^), is capable of selectively damaging dopaminergic neurons of the substantia nigra pars compacta resulting in a decrease of striatal dopamine (DA) level characteristic of PD [21, 22, 23, 24, 25, 26, 27, 28, 29, 30, 31, 32, 33, 34, 35, 36]. The C57Bl/6 mice serve as one of the most sensitive mouse strains regarding MPTP toxicity [37, 38]. In addition to the demonstration of increased sensitivity to neurotoxicity with aging, several studies assessed gender differences in C57Bl/6 mice following MPTP intoxication as well [24, 25, 26, 27, 28, 29, 32, 36, 39, 40, 41, 42, 43, 44]. Although the obtained results are controversial, the majority of studies demonstrated increased sensitivity in males, especially regarding nigrostriatal injury [28, 32, 36, 39, 41, 42, 43, 44]. The reason behind this phenomenon has not been exactly revealed, yet.

In line with the human data demonstrated above, the assessment of neuroprotection in C57Bl/6 male or female mice applying αT supplementation in the MPTP model of PD yields controversial results as well [21, 22, 34, 45, 46, 47, 48, 49, 50, 51]. In summary, only the administration of considerably high doses of αT provided neuroprotective effects only in a portion of studies [45, 46, 48, 50, 51].

In addition to exogenous αT supplementation, another strategy may be the achievement of neuroprotection via the manipulation of endogenous αT homeostasis. The dietary or genetic depletion of brain αT levels yielded conflicting results as well [49, 50]. MPTP intoxication following prolonged dietary vitamin E depletion resulted in increased susceptibility to damage in the substantia nigra, but not in the striatum [49, 52]. On the contrary, genetic vitamin E deficiency (utilizing αT transfer protein (α-TTP) knockout mice) did not influence the striatal DA depletion following MPTP treatment, whereas the number of tyrosine hydroxylase positive neurons of the substantia nigra was not altered at all [50]. These conflicting findings may be partially explained by the differences in the MPTP treatment regimen as well. Our previous study demonstrated that a female-predominant increase in endogenous striatal αT level evolves with aging, providing significant differences between genders already at 16 weeks of age [2].

In light of the available literature data, the aim of the current study was to further confirm the decreased sensitivity of female C57Bl/6 mice to MPTP neurotoxicity and to assess whether this difference is related to elevated endogenous striatal αT content.

## 2 Materials and methods

### 2.1 Materials

The reagents for αT high performance liquid chromatography (HPLC) measurement has already been reported [2], and besides those, we used the following chemicals in this study: disodium-ethylenediaminetetraacetate dihydrate (Na_2_EDTA∗2H_2_O; Lach-Ner s.r.o, Neratovice, Czech Republic), sodium-metabisulfite (Na_2_S_2_O_5_; Fluka, Budapest, Hungary, Honeywell Group), sodium-dihydrogenphosphate (NaH_2_PO_4_; Reanal Laboratory Chemicals, Budapest, Hungary), HPLC purity absolute ethanol (EtOH) and acetonitrile (ACN; VWR International, Radnar, PA, USA), MPTP hydrochloride (MedChemExpress, Monmouth Junction, NJ, USA) and the following substances were obtained from Sigma-Aldrich (Saint Louis, MO, USA): perchloric acid (HClO_4_), phosphoric acid (H_3_PO_4_), sodium hydroxide (NaOH), buthyl-hydroxy-toluol (BHT), sodium-octyl-sulphate (NaOS), 3,4-dihydroxyphenylacetic acid (DOPAC), 3,4-dihydroxybenzylamine hydrobromide (DHBA∗HBr, internal standard (IS)), homovanillic acid (HVA), dopamine hydrochloride (DA∗HCl) and isoproterenol hydrochloride (IPR∗HCl, IS).

### 2.2 Animals

For this study, we utilized C57Bl/6 mice, housed under standard laboratory conditions with free access to food and water. We examined four groups of animals consisting of control and MPTP-treated 16 weeks old male and female mice (initially n = 15 in each group). All animal experiments were carried out in accordance with the Scientific Ethics Committee for Animal Research of the Protection of Animals Advisory Board (XXIV./352/2012.) and were approved by the Committee of Animal Research at the University of Szeged (XI./243/2019.). The required sample size per groups was determined by power analysis (GPower software) with a result of n = 13 per group. Considering the fact that MPTP treatment may result in death in some proportion of animals [30], we decided to increase the sample size to 15 in each group considering the possible mortality rate.

### 2.3 Treatment and sample handling

MPTP hydrochloride was freshly dissolved in saline (pH adjusted to 7.4 with 0.1 M NaOH) and was administered intraperitoneally (i.p.). Male and female mice were randomly divided into 2x2 groups. Two groups received i.p. injection of 12 mg/kg body weight MPTP 5 times at 2 h intervals. The other 2 groups served as controls and received i.p. saline injection 5 times at 2 h intervals. After the last MPTP injection, two male and one female mice were found dead. Regarding the control groups one female mouse was excluded from the study due to unexpected behavior.

One week following the last i.p. injection, all the animals were deeply anesthetized with isoflurane (Forane®; Abbott Laboratories Hungary Ltd., Budapest, Hungary). Sample collection and preparation was similar as described previously [2]. Briefly, plasma and halved striatal samples were collected for the determination of αT and catecholamine concentrations.

Before DA, DOPAC and HVA measurements, the halved striatal samples were weighed and sonicated in ice-cold solution (60 μL/mg striatum) containing 400 μM Na_2_S_2_O_5_, 500 μM Na_2_EDTA∗2H_2_O, and ISs (50 ng/mL DHBA and 200 ng/mL IPR in 334 mM HClO_4_). The samples were centrifuged at 4 °C for 30 min at 12000 RPM, and after the supernatants were collected, 10 μL was injected into the HPLC.

### 2.4 Chromatographic conditions

For the quantification of αT and the IS (rac-tocol) a previously published method was used for both mouse plasma samples (applying diode-array detector (DAD)) [2, 7] and mouse brain samples (fluorescence detector (FLD)) [2]. For the analysis Agilent 1100 HPLC system (Agilent Technologies, Santa Clara, CA, USA) was used under isocratic conditions.

However, for DA, DOPAC and HVA measurements, our previously applied method [35] was modified. Regarding this improved method, the validation process was carried out again on striatal samples. The developed method is applicable for the simultaneous determination of L-DOPA, norepinephrine, 3-methoxytyramine, 5-hydroxyindoleacetic acid, 5-hydroxytryptamine and another IS, 5-hydroxy-N-ω-methyltryptamine as well, but this opportunity was not utilized in the current study.

The mobile phase consisted of 2.20 mM NaOS, 75 mM NaH_2_PO_4_, 100 μM Na_2_EDTA∗2H_2_O and 6.25 v/v% ACN. The pH value was set to 3.0 with 85 w/w% H_3_PO_4_. The mobile phase was delivered at a rate of 1.5 ml/min at 40 °C onto the reversed phase column (Zorbax Eclipse Plus C18, 100 × 4.6 mm i.d., 3.5 μm particle size, Agilent Technologies, Santa Clara, CA, USA) after passage through a precolumn (SecurityGuard, 4 × 3.0 mm i.d., Phenomenex Inc., Torrance, CA, USA). Aliquots were injected with the cooling module set to 4 °C and the working potential to +750 mV, using a glassy carbon electrode and an Ag/AgCl reference electrode.

### 2.5 Validation of the applied methods

All validation processes were carried out with the guidance of ICH and FDA [53, 54, 55]. The following parameters were determined: linearity ranges, limit of detection, limit of quantification, recoveries at three concentration levels, and intra- and interday precisions.

Calibrators were prepared in acidic solution, containing ascorbic acid and BHT, due to the stability issues of some compounds [35], then they were arranged in five different concentration levels. The peak area response ratios were plotted as a function of the corresponding concentration and linear regression computations were evaluated by the least square method with the freely available R software (R Development Core Team, https://www.r-project.org/). Good linearity (R^2^ ≥ 0.99) was detected throughout the concentration ranges for all compounds.

### 2.6 Statistical analysis

All statistical calculations were performed with the use of above-mentioned R software. First, we checked the distribution of data populations with the Shapiro-Wilk test and we also performed the Levene test to confirm the homogeneity of variances. As the distribution proved to be Gaussian and the variances were equal, two-way ANOVA was applied with Tukey HSD *post hoc* test for pairwise comparisons. We decided *a priori* that the comparisons of control and treatment groups with opposite gender may not yield meaningful information, and accordingly, only four comparisons were implemented regarding the four groups. In case of model construction, ANCOVA was applied. We rejected the null hypothesis when the *p*-values were <0.05, and in such cases the differences were considered significant. If any significant change was observed, the effect size was calculated (omega square (ω^2^) for two-way ANOVA, partial eta square (p. η^2^) for ANCOVA and *Cohen's d* for Tukey HSD). Pearson correlation analysis with *post hoc* Bonferroni correction for the number of analyses was used to investigate the possible relationship between endogenous striatal αT content and DA level in each group. Data were plotted as means (±S.D.). The measured values were presented in ng/mg wet weight (ww) and nmol/g ww regarding catecholamines and αT, respectively, to allow comparison with previous results of the authors [2, 35].

## 3 Results

### 3.1 HPLC measurement of DA, DOPAC and HVA

**Figure 1:**
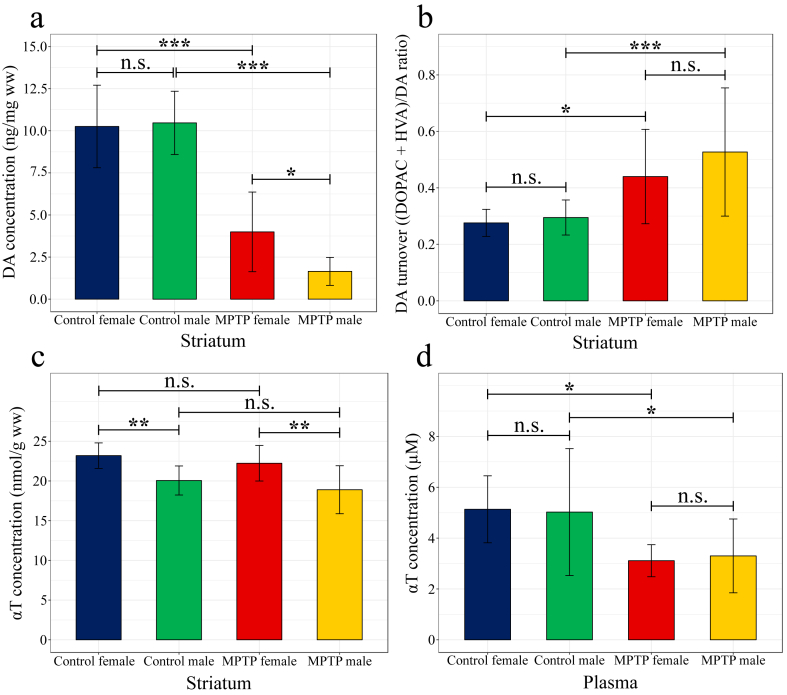
Measured analyte concentrations in mouse striatal and plasma samples. DA levels (a), DA turnover (b), αT levels in the striatum (c) and in the plasma (d) are presented for the demonstration of the effect of MPTP treatment. Data are presented as mean (±S.D.); n (control and MPTP-treated females) = 14; n (control males) = 15; n (MPTP-treated males) = 13; *αT* α-tocopherol; *DA* dopamine; *DOPAC* 3,4-dihydroxyphenylacetic acid; *HVA* homovanillic acid; *MPTP* 1-methyl-4-phenyl-1,2,3,6-tetrahydropyridine; *n.s.* not significant; ∗*p* < 0.05; ∗∗*p* < 0.01; ∗∗∗*p* < 0.001.

The measurements of DA, DOPAC and HVA concentration from striatal samples are presented in Table 1 and Figure 1a. The implementation of two-way ANOVA with Tukey HSD *post hoc* test yielded the following results.

**Table 1:** DA, DOPAC and HVA concentration levels (ng/mg ww) and DA turnover in the striatum of mice.

Analytes or ratio	Control females^1^	Control males^2^	MPTP females^1^	MPTP males^3^
DA	10.255 ± 2.451	10.692 ± 1.983	3.995 ± 2.364	1.651 ± 0.830
DOPAC	0.989 ± 0.170	1.097 ± 0.273	0.496 ± 0.248	0.195 ± 0.135
HVA	1.781 ± 0.453	1.938 ± 0.696	1.039 ± 0.458	0.604 ± 0.388
(DOPAC + HVA)/DA	0.276 ± 0.048	0.295 ± 0.062	0.440 ± 0.167	0.527 ± 0.227

Data are presented as mean (±S.D.); ^*1*^n = 14; ^*2*^n = 15; ^*3*^n = 13; *DA* dopamine; *DOPAC* 3,4-dihydroxyphenylacetic acid; *HVA* homovanillic acid; *MPTP* 1-methyl-4-phenyl-1,2,3,6-tetrahydropyridine; *ww* wet weight.

Significant differences were observed in DA levels regarding treatment (F (1, 52) = 196.355, *p* < 0.001, ω^2^ = 0.2201) and regarding treatment *vs.* gender as well (F (1, 52) = 5.703, *p* < 0.05, ω^2^ = 0.0053), but not for gender (F (1, 52) = 3.627, *p* = 0.062, ω^2^ = 0.0033). *Post hoc* analysis with Tukey HSD test yielded significantly decreased DA concentrations in MPTP-treated *vs.* control females (*p* < 0.001, Figure 1a), with an effect size of -2.600, in MPTP-treated *vs.* control males (*p* < 0.001, Figure 1a) with an effect size of -5.925, and in MPTP-treated males *vs.* females (*p* < 0.05, Figure 1a) as well with an effect size of -1.303.

Similar to the above-mentioned changes in DA levels, significant differences were observed in DOPAC levels as well, regarding treatment (F (1, 52) = 143.741, *p* < 0.001, ω^2^ = 0.1769) and regarding treatment *vs.* gender (F (1, 52) = 12.481, *p* < 0.001, ω^2^ = 0.0141), but not for gender (F (1, 52) = 2.373, *p* = 0.129, ω^2^ = 0.0022). *Post hoc* analysis with Tukey HSD test yielded significantly decreased DOPAC concentrations in MPTP-treated *vs.* control females (*p* < 0.001), with an effect size of -2.309, in MPTP-treated *vs.* control males (*p* < 0.001) with an effect size of -4.094 and in MPTP-treated males *vs.* females (*p* < 0.01) with an effect size of -1.491.

Significant differences were observed in HVA levels as well regarding treatment (F (1, 52) = 59.920, *p* < 0.001, ω^2^ = 0.1137) and regarding treatment *vs.* gender (F (1, 52) = 5.704, *p* < 0.05, ω^2^ = 0.0090), but not for gender (F (1, 52) = 0.464, *p* = 0.499, ω^2^ = -0.0008). *Post hoc* analysis with Tukey HSD test yielded significantly decreased HVA concentrations in MPTP-treated *vs.* control females (*p* < 0.01), with an effect size of -1.629, and in MPTP-treated *vs.* control males (*p* < 0.001) with an effect size of -2.464.

A metabolite rate was also determined in all the four groups and the values were compared with two-way ANOVA and Tukey HSD *post hoc* test. There was a significant increase in the calculated (DOPAC + HVA)/DA ratio (DA turnover) regarding the treatment (F (1, 52) = 26.129, *p* < 0.001, ω^2^ = 0.0538), but not for gender (F (1, 52) = 1.842, *p* = 0.181, ω^2^ = 0.0019) and treatment *vs.* gender (F (1, 52) = 0.779, *p =* 0.382, ω^2^ = -0.0004). The Tukey HSD *post hoc* test yielded significantly increased DA turnover in MPTP-treated *vs.* control females (*p* < 0.05; Figure 1b) with an effect size of 1.335, and in MPTP-treated *vs.* control males (*p* < 0.001; Figure 1b) with an effect size of 1.438.

### 3.2 HPLC measurement of αT

The results of the measurements of αT concentration from plasma and striatal samples are presented in Table 2, Figure 1c and d. The applied two-way ANOVA demonstrated significant difference in αT level of plasma regarding treatment (F (1, 52) = 18.227, *p* < 0.001, ω^2^ = 0.0396), but not for gender (F (1, 52) = 0.006, *p* = 0.938, ω^2^ = -0.0023) and gender *vs.* treatment (F (1, 52) = 0.115, *p* = 0.736, ω^2^ = -0.0020). *Post hoc* analysis with Tukey HSD test yielded significantly decreased αT concentrations in MPTP-treated *vs.* control females (*p* < 0.05, Figure 1d), with an effect size of -1.958, and in MPTP-treated *vs.* control males (*p* < 0.05, Figure 1d) with an effect size of -0.829.

**Table 2:** αT concentration levels in the plasma and the striatum of mice.

	Plasma (μM)	Striatum (nmol/g ww)
Control females^1^	5.14 ± 1.32	23.19 ± 1.61
Control males^2^	5.03 ± 2.50	20.06 ± 1.83
MPTP females^1^	3.11 ± 0.63	22.23 ± 2.24
MPTP males^3^	3.30 ± 1.45	18.90 ± 3.02

Data are presented as mean (±S.D.); ^*1*^n = 14; ^*2*^n = 15; ^*3*^n = 13; *αT* α-tocopherol; *MPTP* 1-methyl-4-phenyl-1,2,3,6-tetrahydropyridine; *ww* wet weight.

Regarding the striatum, there was a significant difference for gender (F (1, 52) = 29.680, *p* < 0.001, ω^2^ = 0.0055), but not for treatment (F (1, 52) = 2.543, *p* = 0.117, ω^2^ = 0.0004) and for treatment *vs.* gender (F (1, 52) = 0.029, *p* = 0.865, ω^2^ = -0.0002). The Tukey HSD *post hoc* test revealed significantly higher αT concentrations in control female *vs.* male mice (*p* < 0.01, Figure 1c), and in MPTP-treated female *vs.* male mice as well (*p* < 0.01, Figure 1c) with effect sizes of 1.811 and 1.261, respectively.

The results of the assessment of the relationship between DA and αT levels in the striatum are presented in the Supplementary Material.

## 4 Discussion

PD is the second most common neurodegenerative disorder with an increasing prevalence in the aging population and in males [56]. These phenomena, i.e., increasing sensitivity to nigrostriatal injury with aging and in males have been considerably well represented in the MPTP mouse model of PD as well [28, 32, 36, 39, 41, 42, 43, 44]. Although the possible role of sexual hormones behind these findings was proposed by several studies [39, 41, 57], the exact explanation behind gender differences is still missing.

Amongst strategies of ameliorating disease progression, a popular approach may be the reduction of oxidative injury characteristic of PD [1]. Probably the most exhaustive trial to achieve this aim via the administration of αT was the DATATOP study, but the results did not support any neuroprotective effect [9]. Although literature data, coming from preclinical and human studies, are controversial regarding this topic, it can be proposed that the prolonged application of high dose αT initiated in early phases may have beneficial effects on the neurodegenerative processes [18].

The authors demonstrated in one of their previous studies that a significant rise of striatal αT evolved with aging, more pronounced in female mice and already significant at 16 weeks of age [2]. These phenomena may be explained by that the aging brain tries to increase its antioxidant capacity, predominantly in females, which may provide an enhanced protection against neurodegeneration. However, this hypothesis, i.e., whether higher striatal αT level in females correlates with less reduction in striatal DA level following MPTP intoxication, has never been tested before. Accordingly, the aim of the current study was to examine whether gender-related difference in endogenous striatal αT level has an influence on the distinctly decreased DA levels in MPTP-treated C57Bl/6 female and male mice. The results demonstrated that striatal DA levels of MPTP-treated female and male mice were significantly decreased to 39% and to 15.4%, respectively, compared to the corresponding control groups. The significantly decreased sensitivity to MPTP intoxication in female C57Bl/6 mice compared to their male counterparts is in line with the majority of literature data [28, 39, 41, 42, 43, 44]. It was also assessed whether these findings may be related to differences in DA turnover. Although DA turnover significantly increased in MPTP-treated mice compared to controls, which also corresponds to the results of other studies [58, 59], no difference between genders could be demonstrated. Regarding striatal αT levels, the findings of the current study confirmed our previous results [2], i.e., the concentrations were significantly higher in the striata of females compared to males already at 16 weeks of age. Surprisingly, but in line with previous findings [49], these striatal αT levels were not influenced by MPTP treatment, however, plasma αT levels significantly decreased in both genders. Keeping in mind that the samples for bioanalytical studies were obtained 7 days following acute MPTP intoxication, a peripheral to central redistribution might took place as an effort to prevent brain injury. In the next part of the study the possible relationship between the above-detailed 2 parameters were assessed, i.e., whether higher striatal αT content is capable of exerting protection against MPTP-induced neurotoxicity. However, the applied statistical analyses could not demonstrate any significant correlation between striatal DA and αT levels following MPTP treatment (Supplementary Material), and therefore, the hypothesis that higher striatal αT concentration in females may be responsible for the less reduction in striatal DA level following MPTP intoxication at 16 weeks of age could not be proved. Nevertheless, the finding itself that endogenous striatal αT content does not seem to be a major player against MPTP-induced deteriorations may be supported by the data obtained from studies with α-TTP deficient mice or with the application of dietary restriction [49, 50]. Even α-TTP −/− mice with essentially undetectable level of brain αT were not more prone to MPTP-related striatal DA decrease compared to wild-type controls [50].

Limitations of the current study include the lack of its extension for the assessment of the relationships between striatal DA and αT levels in further age groups. However, the authors presume that although both the sensitivity to MPTP treatment [41, 42, 44] and the striatal level of αT [2] increase with aging with the enlargement or at least the persistence of the above-mentioned differences between genders, their relationship does not likely change. Accordingly, keeping in mind the 3R principle (replacement, reduction and refinement) of animal experiments as well, this extension was out of scope of the present work.

In conclusion, the current study was the first to examine the possible role of elevated αT in female C57Bl/6 mice behind their decreased sensitivity to MPTP intoxication. The results, i.e., no significant correlation was found between the above two parameters, may further confirm that αT does not play a major role against neurotoxicity induced by MPTP. Anyway, the assessment of factors behind the decreased sensitivity of female mice to nigrostriatal MPTP toxicity may warrant further studies to explore novel possible therapeutic targets.

## Declarations

### Author contribution statement

Nikolett Nánási, Edina K Cseh, Diána Martos, Levente Hadady: Performed the experiments; Analyzed and interpreted the data; Wrote the paper.

Gábor Veres: Analyzed and interpreted the data; Contributed reagents, materials, analysis tools or data; Wrote the paper.

Péter Klivényi, László Vécsei: Conceived and designed the experiments; Contributed reagents, materials, analysis tools or data; Wrote the paper.

Dénes Zádori: Conceived and designed the experiments; Analyzed and interpreted the data; Contributed reagents, materials, analysis tools or data; Wrote the paper.

### Funding statement

This work was supported by GINOP-2.3.2-15-2016-00034, EFOP-3.6.1-16-2016-00008, the 10.13039/501100005881Ministry of Human Capacities, Hungary (20391-3/2018/FEKUSTRAT) and the Hungarian Brain Research Program (2017-1.2.1-NKP-2017-00002 NAP VI/4).

Dénes Zádori was supported by the János Bolyai Research Scholarship of the Hungarian Academy of Sciences and by the UNKP-18-4 New National Excellence Program of the Ministry of Human Capacities.

Edina K Cseh was supported by the UNKP-19-3 New National Excellence Program of the Ministry for Innovation and Technology and EFOP-3.6.3-VEKOP-16-2017-00009.

Open access funding was provided by the University of Szeged (SZTE) (4467).

### Competing interest statement

The authors declare no conflict of interest.

### Additional information

No additional information is available for this paper.
