# Supplementary material for: The assessment of possible gender-related effect of endogenous striatal alpha-tocopherol level on MPTP neurotoxicity in mice
Source: Heliyon. 2020 Jul 11;6(7):e04425. doi: 10.1016/j.heliyon.2020.e04425 (PMC7358721; doi:10.1016/j.heliyon.2020.e04425)
Supplement: Supplementary file 1 — Supplementary Material.pdf [file mmc1.pdf]

Supplementary Material

The assessment of possible gender-related effect of endogenous striatal alpha-tocopherol level on MPTP neurotoxicity in mice

Nikolett Nánási[1], Gábor Veres[1,2], Edina K Cseh[1], Diána Martos[1], Levente Hadady[1], Péter Klivényi[1], László Vécsei[1,2], Dénes Zádori[1]*

[1]Department of Neurology, Interdisciplinary Excellence Center, Faculty of Medicine, Albert Szent-Györgyi Clinical Center, University of Szeged, Szeged, Hungary

[2]MTA-SZTE Neuroscience Research Group, Szeged, Hungary

Study of possible relationships between DA and αT levels in the striatum

Regarding the assessment whether endogenous α-tocopherol (αT) content could affect the change in dopamine (DA) levels following 1-methyl-4-phenyl-1,2,3,6-tetrahydropyridine (MPTP) treatment, data were analyzed by ANCOVA. The results of this complex statistical analysis demonstrated that MPTP treatment significantly influence striatal DA level (F (1, 52) = 8.689, $p < 0.01$, partial η^2 (p. η^2) = 0.761), but striatal αT level did not have a significant influence on either striatal DA level (F (1, 52) = 0.487, $p = 0.488$, p. $\eta^2 = 0.007$) or on its decrease following MPTP treatment (assessment of interaction; F (1, 52) = 1.879, $p = 0.176$, p. $\eta^2 = 0.035$). For the confirmation of these findings in each group, correlation analyses were carried out, similarly presenting no significant correlation between striatal DA and αT levels (Pearson's R^2s and p values prior to Bonferroni correction: control females: 0.0069 and 0.779, respectively; control males: 0.036 and 0.495, respectively; MPTP-treated females: 0.023 and 0.603, respectively; MPTP-treated males: 0.369 and 0.028, respectively; Supplementary Fig. 1).

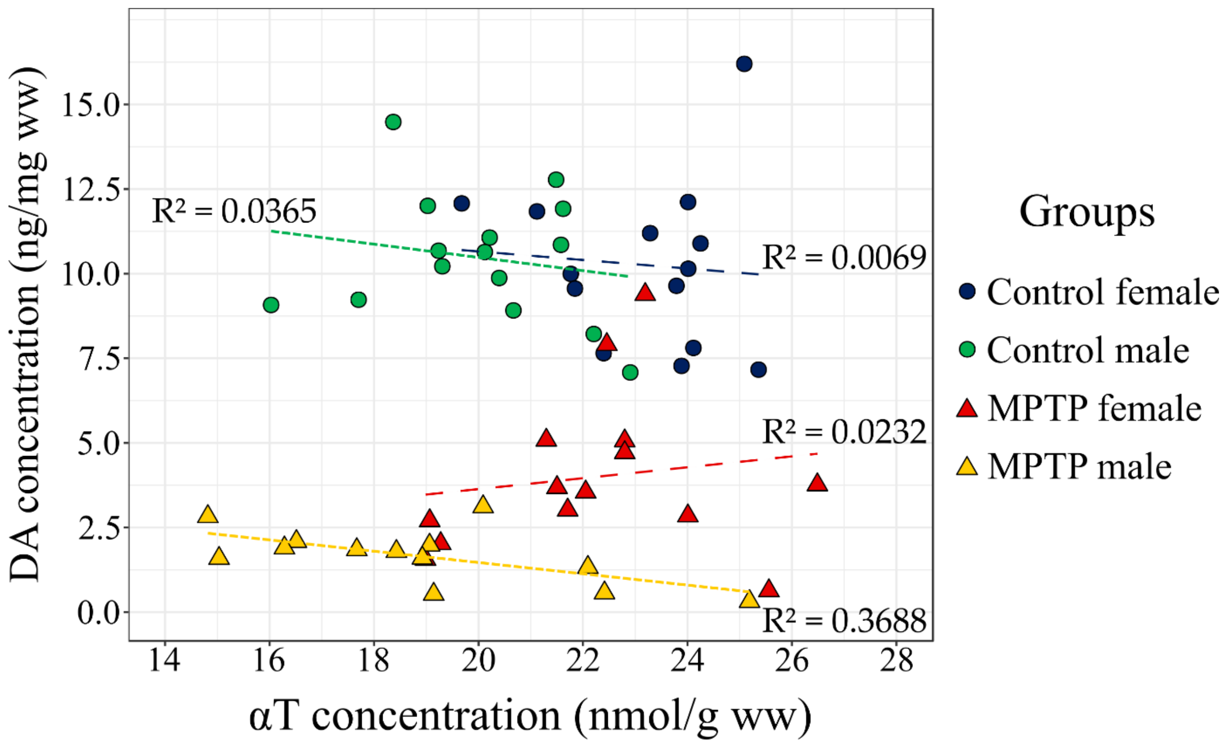

Supplementary Fig. 1. Possible correlations between endogenous αT concentrations and DA levels in the striatum. Following Bonferroni correction, no statistically significant correlations were demonstrated. n (control and MPTP treated females) = 14; n (control males) = 15; n (MPTP treated males) = 13; *αT* α-tocopherol; *DA* dopamine; *MPTP* 1-methyl-4-phenyl-1,2,3,6-tetrahydropyridine.
